# Similarity and Positivity of Personality Profiles Consistently Predict Relationship Satisfaction in Dyads

**DOI:** 10.3389/fpsyg.2018.01009

**Published:** 2018-06-29

**Authors:** Hermann Brandstätter, Veronika Brandstätter, Rainer B. Pelka

**Affiliations:** ^1^Institute of Education Science and Psychology, Johannes Kepler University Linz, Linz, Austria; ^2^Department of Psychology, University of Zurich, Zurich, Switzerland; ^3^Institute for Applied Statistics, Munich, Germany

**Keywords:** personality, similarity, close relationships, dyads, suppressor variables, actor–partner- interdependence-model, structural equation modeling

## Abstract

The effect of similarities in the personality traits of romantic partners on their relationship satisfaction (*RS*) has often been studied, albeit with mixed results. Beyond the main effects of personality traits, incremental validity was often completely missing, or at least very low. In contrast, our five studies, three cross-sectional – including one study on leader–follower dyads to secure generalizability – and two longitudinal, show that, in predicting *RS*, the beta-coefficients of *distance* (where distance is defined as the average across items of absolute differences between the two partners’ self-ratings) or *positivity* (where positivity is defined as the frequency of extremely positive self-ratings) increase when either the *positivity* of the profiles or the *distance* between the profiles is added as second predictor. Thus, positivity and distance seem to function as reciprocal suppressor variables that allow controlling for irrelevant components of the predictors. Consequently, when combined with positivity, distance proved to be a consistently better predictor of *RS* than has been reported in most previous studies. Combining profile distance with profile positivity appears to be promising well beyond research on *RS*, in that an individual profile of traits can be matched with a profile of a specific environment’s offers and demands when person-environment fit is the focus of interest.

## Introduction

Relationship satisfaction (*RS*) is generally perceived as being highly important for human well-being (Myers, 1999) and has for decades been a topic of social psychological research and counseling practice. The positive influence on *RS* of the partners’ socially desirable personality traits is the main effect of positivity, defined as individual frequency of extreme self-ratings. The influence on *RS* of the partners’ (dis)similarity in these traits refers to our second global predictor variable, understood in statistical terms as the interaction effect between the two partners’ self-ratings and calculated as an average across items of the absolute differences between the partners’ self-ratings.

The main effects of personality traits are well documented in the literature (Kenny et al., 2006). Emotional Stability (Neuroticism reversed), Agreeableness, Conscientiousness, and to a lesser degree also Openness and Extraversion, are the Big Five variables that are consistently linked to *RS*. In a meta-analysis, Karney and Bradbury (1995) showed that positive self-ascribed traits tend to predict marital success, whereas negative self-ascribed traits tend to predict marital failure. Thus, it makes sense to look for a global positivity scale encompassing quite diverse self-descriptions of personality traits related to the valuable aspects of human experience and behavior. Such a scale should in turn allow to predict *RS* as well as many other positively valued instances of everyday life.

We expect that positivity in answering a personality questionnaire will be associated with positivity of observed real behavior. In the present paper, we propose to construct a positivity measure that is conceptually related to the construct of core self-evaluation, comprising the positively valued traits of self-esteem, generalized self-efficacy, internal locus of control, and emotional stability (Judge et al., 2003). Both constructs, positivity and core self-evaluation, share the idea of a higher order personality factor encompassing the valence of lower order personality traits and allowing non-trivial predictions of subjective experience (e.g., happiness in the relationship) or objectively observable behavior (e.g., separation or divorce).

A second approach to a kind of core self-evaluation is presented by Caprara et al. (2012). These authors understand the latent construct of positivity as higher order factor, with self-esteem, life satisfaction, and optimism as observable indicators. In their reports high positivity, defined as the tendency to view life and its experiences with a positive outlook (Caprara et al., 2012), was associated with many positively valued outcomes.

Our positivity measure is based on the PASK5 (Brandstätter, 1988, 2012). The PASK5 consists of 32 bipolar adjective pairs each of which represents one of the 16PF primary dimensions (Cattell and Schuerger, 2003). Sample items are “well-balanced—irritable,” “self-doubting—self-assured,” “restrained—lively,” “impersonal—outgoing.” In a series of analyses these adjectives have also proved as reliable predictors of the five global factors of the NEO-PI-R (Ostendorf and Angleitner, 2005). Our positivity measure is conceptually related to the scales of Judge et al. (2003) and Caprara et al. (2012), because both constructs share – together with our positivity measure – the rationale of a higher order personality factor encompassing the valence aspects of lower order traits. However, in the case of PASK5 the profiles are built on the level of 32 items, whereas the profiles of Judge et al. (2003) and Caprara et al. (2012) are based on aggregated scales. The core self-evaluation of Judge et al. (2003) as well as the positivity construct of Caprara et al. (2012) had conceptually proved as sufficiently similar to our positivity measure that we can derive comparable hypotheses predicting *RS*. It is not our intention, though, to review the literature on higher order personality dimensions, but to show that our similarity-positivity-model of *RS* can be integrated into a wider theoretical perspective.

Besides positivity as a predictor of *RS*, we need to deal with the distance (dissimilarity) between the partners’ profiles. The theoretical relationship between distance and *RS* is more complicated than that between positivity and *RS*, as we will see in more detail below. Keep in mind, that positivity and distance (dissimilarity of personality profiles) are the only explanatory variables in our model of dyadic *RS*.

Many studies report positive effects of similarity on *RS*, in agreement with popular beliefs, (for instance, Russell and Wells, 1991; Acitelli et al., 2001; Luo and Klohnen, 2005; Gaunt, 2006; Gonzaga et al., 2007; Luo et al., 2008; Decuyper et al., 2012). However, a number of studies do not confirm the assumed positive effects of similarity. In particular, controlling for the main effects of traits involved in the similarity measures tends to attenuate, if not completely absorb similarity effects (Kenny and Acitelli, 1994; Watson et al., 2004; Barelds, 2005; Montoya, 2008; Dyrenforth et al., 2010; Altmann et al., 2013; Becker, 2013; Furler et al., 2013; Tidwell et al., 2013; Wood and Furr, 2016). Adding a second predictor in a multiple regression model may increase, affect not at all or decrease the coefficient of the first one. Only in the first case, the second predictor is a suppressor. In the third case we may speak of a redundant predictor which means that adding the second predictor makes the first superfluous. To understand that controlling for positivity can increase or decrease similarity effects, one needs to integrate the suppressor effect.

Considering the inconsistent empirical evidence, it is worth taking a closer and novel, methodologically oriented look at the influence of global similarity on *RS*. Notwithstanding the conflicting empirical evidence, we are putting forward the hypothesis, to be tested in five studies, that distance predicts *RS* to a substantial degree. Remember that distance is operationalized as the mean absolute difference between the partners’ self-ratings with respect to 32 bipolar descriptive adjectives (Brandstätter, 2012). Positivity is operationalized as the individual frequency of extreme positive self-ratings on bipolar scales. Because measures of similarity are commonly confounded with measures of positivity (see, for instance, Wood and Furr, 2016), we must from the start think about the combined effects of distance and the two partners’ positivity scores.

Before we consider various ways of measuring similarity and their connection with the positivity of the ratings, we simply ask: Why should partners with similar personality traits feel better in their relationship than dissimilar ones? These are our theoretical reasons for the assumption of a positive similarity balance.

### Similar Emotions

Gonzaga et al. (2007) show that partners who are similar in their self-ratings of personality traits experience similar emotions in many situations, facilitating understanding each other’s spontaneous and intuitive responses to circumstances and events in their common environment (Shiota et al., 2006).

### Similarity in Coping With Stress

According to the Vulnerability Stress Adaptation Model (Karney and Bradbury, 1995) personality traits are distant causes of behavior. In the context of stress research, personality traits are understood as vulnerabilities (in experiencing events as more or less stressful) and as specific skills (needed in coping with and adapting to stressful situations). When the partners agree in perceiving events or circumstances as stressful and in how to cope with stress, it is easier to avoid and to overcome tensions that could impair *RS*.

### Similarity in Beliefs

At the level of reflected reasoning (for a comparison of intuitive and deliberate judgment, see Gigerenzer, 2007; Kruglanski and Gigerenzer, 2011), partners with a high degree of similarity will support each other’s world view and beliefs (Morry and Graines, 2005). They find themselves in situations of misunderstanding and conflict of values, attitudes and intentions less often, possibly due to congruent use of heuristics in judgment and decision making. Consequently, they will be better able to develop a mutually satisfying relationship.

### Similarity and Equity

Similarity implies equity, i.e., the subjective value of resources given and received is well balanced (Austin and Walster, 1974). Thus, even being similar in negative traits can have some merits as, for instance, equality in physical attractiveness is a moment of stability for the partnership (White, 1980).

### Perceived Similarity and Cognitive Consistency

Perceived similarity is both a cause and an effect of liking (Morry and Graines, 2005; Montoya, 2008; Morry et al., 2011). When *RS* is high (for some other reason than similarity), perceiving the partner as being similar is *cognitively consistent* (Simon et al., 2015), therefore rewarding and thus contributing to a positive link between similarity and *RS*.

### Positive Effects of Dissimilarity: Variety and Complementarity

The generally assumed positive effect of similarity on *RS* has been questioned by Shiota and Levenson (2007), who present data from middle aged couples with positive effects of *dis*similarity on *RS*. The authors explain their unexpected findings that dissimilar partners provide a stimulating variety of experiences to each other and develop a kind of specialization in coping with their daily life tasks both of which foster *RS*. We have to consider a specific form of dissimilarity, that is, complementarity: the partners’ opposite characteristics provide satisfaction by compensating each other’s weaknesses (e.g., Markey and Markey, 2007). For example, take the case of a couple where one partner is dominant while the other is submissive (Markey et al., 2010). Presumably, this form of complementarity prevents a couple from constant conflict imminent in a relationship with two highly dominant partners. Dominance-submissiveness, though, is one of the few dimensions where *dis*similarity is related to positive outcomes in a social interaction (e.g., Tiedens and Fragale, 2003). Actually, the literature provides many more instances of negative effects of dissimilarity than positive effects of complementarity. Thus, it is reasonable to assume that a score of global dissimilarity as given with distance in our studies generally will be correlated with negative experiences in social relationships (see **Appendix** for an illustration of the trivariate relationship).

## Preliminary Methodological Considerations

All five studies that will be reported here use the same personality questionnaire (i.e., PASK5, see below), share distance combined with our measure of positivity of personality profiles as a novel combination of variables predicting *RS*, and apply structural equation modeling (SEM) to test the fit between theoretical and empirical variance-covariance structures. We will therefore begin by reflecting on some methodological issues, which are relevant to the entire set of studies, before presenting the individual studies in detail. In order to facilitate comprehension for the reader, we have included some redundancy in clarifying statistical issues, in particular with respect to measuring positivity and distance.

### Personality Adjective Scales PASK5

Originally developed as proxies for Cattell’s 16PF (Brandstätter, 1988; Cattell and Schuerger, 2003), the scales were later adapted as short substitutes for the five global scales of the German version of the NEO-PI-R (Ostendorf and Angleitner, 2005; Brandstätter, 2012). In our studies, however, the focus is not on the five global scales, but on the item-based personality profiles, specifically on their global positivity and on the global (dis)similarity between the two partners’ personality profiles. In the person-centered approach, the larger number of scales – 32 items instead of 5 scales – has the advantage of a higher reliability of the (dis)similarity and positivity measures and of a higher completeness of the trait facets included. Although, completing the PASK5 questionnaire only takes about 7 min, internal consistency, stability, and construct validity of the PASK5 are very similar to those of the NEO-PI-R (cf. Brandstätter, 2012).

### Positivity of Personality Profiles

An important characteristic of the self-ratings of personality traits is their positivity or social desirability. One can hardly speak of positivity in rating personality traits without referring to response sets, such as acquiescence or social desirability. Response sets are commonly treated as a nuisance in personality assessment. Not so by Ferrando et al. (2009) who have conceptualized a participant’s socially desirable responses as valuable information for predicting and explaining behavior in social situations. Adopting this approach, we assume that positivity of personality self-ratings is a summary of sufficiently realistic self-attributions of valuable personality traits.

There are, of course, various ways of assessing the positivity of a personality profile^1^. For example, experts could judge the positivity of the items. Weighting the participants’ responses to the items with the experts’ social desirability scores for the items and aggregating the weighted responses would result in a reasonable global positivity measure. Alternatively, one could simply add the Big Five values (with reversed scoring for Neuroticism) to produce a composite of positive personality characteristics. In contrast, our measure of positivity follows a different strategy, taking into account the fact that people tend to give more or less positive self-ratings to their personality traits. Individual differences in positivity of self-ratings are an important personal characteristic. The more extreme the ratings, the more positive the traits of the rater. Consequently, our positivity measure is based on the distribution of answers on 9-point bipolar rating scales for value-laden traits. Such a distribution is usually highly skewed, positively when the left pole is positive (e.g., *well-balanced* versus *irritable*) or negatively, when the right pole is positive (e.g., *gets worried easily* versus *emotionally stable*). Thus, the individual frequency of extreme scores (1, 2, 8, 9) provides a reasonable measure of positivity, not seriously disrupted by rare cases of extreme negative self-attributions. In our case, scores higher than two *and* lower than eight are neglected when calculating the positivity measure^2^.

Due to the design of the PASK5 and its use in characterizing oneself, extremity means positivity and is, as a consequence of the scale design, highly correlated in the pilot study (*r* = 0.95, *n* = 1183) with the intra-individual scatter (standard deviation) of a person’s responses to the 32 profile items, which would justify using positivity and scatter as equivalent measures.

Our positivity measure is peculiar in that persons with the same positivity score can differ widely in its composition. One might excel with items indicating Agreeableness, another with items in the domain of Conscientiousness, a third with items rooted in Extroversion, etc. Therefore, participants themselves determine what kind of experience or behavior they perceive as positive or negative by giving specific items extreme (i.e., positive), others only moderate to low ratings. Such an idiosyncratic definition of positivity may be part of its expected success in predicting *RS*. Because people generally tend to self-attribute positive traits, the PASK5 and similar questionnaires tell us what kinds of behavior and subjective experience are judged on average as being positive and also how positive a participant perceives herself/himself to be.

In the perception of people, counting the daily frequencies of events, such as friendly acts in a partnership, can be more relevant and informative for judging the quality of a relationship than averaging scores on a Likert scale. In this context, Kruglanski and Gigerenzer (2011, p. 101) state: “To estimate a criterion, do not estimate weights, but simply count the number of positive cues.” A measure of positivity that is based on this heuristic is admittedly unconventional, but possibly a better predictor of *RS* than alternative measures, at least when PASK5 or a similar questionnaire is used to self-rate personality traits.

Moreover, we can defend our positivity measure by pointing to its reliability and its validity when it is combined with distance in predicting *RS*. In our pilot study on personality aspects of academic performance (with *n* = 1183 students applying to a college of health sciences), the split-half reliability coefficients of distance and positivity (Form *A* and Form *B* of the PASK5) were *r* = 0.64 and *r* = 0.68, corresponding to quite reliable coefficients α = 0.78 and α = 0.81, respectively.

In the same sample, the correlations of positivity with the global scales of the Five Factor Model (FFM) are: Conscientiousness *r* = 0.48, Neuroticism *r* = -0.20, Agreeableness *r* = 0.24, Openness *r* = 0.19, and Extroversion *r* = 0.35. Altogether, in a multiple regression analysis, 57% of the variance of positivity are explained by the Big Five. The positivity variable covers the valence aspects of the PASK5-based global personality dimensions quite well. The Big Five have quite often proved to be valid predictors of *RS*, as reported by Dyrenforth et al. (2010) and Furler et al. (2013), for example. Hence, we can expect that positivity, encompassing the socially desirable aspects of the FFM dimensions, combined with distance, will also be a valid predictor in our studies of *RS*.

### Distance Between Two Personality Profiles

Among the variety of (dis)similarity indices (Kenny and Acitelli, 1994; Kenny et al., 2006; Furr, 2008, chapter 12), we consider McCrae’s (2008) index of profile agreement (*I*_pa_), designed for *z*-transformed variables, to be the most useful for studying a combination of extremity and (dis)similarity effects. It is based on the mean (Σ*M*^2^) and difference (Σ*d*^2^) of the partners’ *z*-transformed ratings and implies moderating the negative effects of distance by giving a bonus to the congruence of extreme scores. This index of profile agreement is described by the following equation:

$$\begin{array}{c} I_{\mathrm{pa}}\;=\;(k\;+\;2\Sigma_{M}{}^{2}\;-\;d^{2})/\surd 10^{*}k \end{array}$$

*k* = number of items, *M* = mean of the two partners’ *z*-scores of item responses, and *d* = signed difference between the two *z*-scores.

Although, inspired by McCrae (2008), we chose a modified approach, (a) separating the distance and extremity components of the profile similarity index and using these components as the only predictor variables in our model, (b) basing the extremity score not on the two partners’ average squared *z*-scores, but on the individual frequency of extreme scores, thus allowing extremity to be interpreted as positivity for the individual profile, and (c) defining distance (*D*) as the average (across the 32 items of the PASK5) of nearly normally distributed absolute differences (not of squared differences, *D*^2^) between two personality profiles. All these adaptations had been proved to work properly in the pilot study.

The global distance measure *D* is a composite of the partners’ differences in elevation (i.e., intra-individual average of scores), shape (i.e., idiosyncratic pattern of an individual’s scores), and scatter (i.e., intra-individual variance of scores) of their personality profiles. In order to keep the complexity of the models at a moderate level, and because their effects on *RS* were assumed to be similar, these components of *D* have not been analyzed separately (see Decuyper et al., 2012 for separate analyses of the components).

### Confounding of Similarity With Positivity

Wood and Furr (2016) point to a special problem that arises with any kind of similarity measure (cf. also Furler et al., 2013). These measures would automatically be linked to *RS*, simply because they would be confounded with the positivity of the ratings. Controlling measures of similarity for positivity of the personality profiles has become a methodological must in recent years, as for example Wood and Furr (2016) have shown for a variety of personality inventories and similarity measures. They speak of a Normative Desirability Confound (NDC), which all kinds of global similarity indices are said to be fraught with.

It is therefore commonly recommended (e.g., Kenny et al., 2006; Dyrenforth et al., 2010; Wood and Furr, 2016) that (dis)similarity effects should be controlled for stereotypes, i.e., for people’s average responses as females and males, or as leaders and followers, by subtracting the average (normative) profile from the individual profiles before calculating the ‘distinctive’ similarity score.

Our solution to the problem is different: Instead of controlling stereotype effects by subtracting the average (normative) profile from the individual profile, we control distance for positivity by including both distance and positivity as predictors of *RS* in our models.

### Suppressor Effects

In the pilot study with students applying for admission to a competitive higher education program, we found that (a) the distance between the individual personality profiles and the collective profile of the accepted students, and (b) positivity of the individual profiles, were valid negative and positive predictors, respectively, of the first semester grade point average. This had been expected; what was unexpected, however – a kind of serendipity – was that distance and positivity served as reciprocal suppressors of irrelevant components of the predictors (for the concept of reciprocal suppressor variables see Maassen and Bakker, 2001). Following this methodological hint from our research on person-environment fit, we stated the hypothesis, which applies to all five of our studies, that including positivity in addition to distance or distance in addition to positivity as predictors of *RS* will enhance the negative β-coefficients of distance and the positive β-coefficients of positivity.

### Comparing and Selecting Alternative Models

Following the advice of Arbuckle and Wothke (1999), or Kenny et al. (2006), we aimed to reduce the complexity of models and secure a higher level of generalizability by setting some parameters equal, for instance, across the social roles of the partners (men vs. women, or leaders vs. followers), if this was both theoretically and statistically reasonable.

### Fit of the Measurement Models

We always tested the fit of measurement models (representing the relationship between the latent constructs and their indicators) before testing the full structural models. For the sake of brevity, we will not report the generally good fit indices of the various measurement models, instead reporting only the fit of the overall models.

In the following reports on our studies, we first present two cross-sectional analyses, one with a convenience sample of students at the University of Zurich (*n* = 60 couples; Study 1) and another with a sample recruited by students of the Johannes Kepler University of Linz among their relatives and acquaintances (*n* = 66 couples). Cross-sectional Study 2 is a close replication of Study 1, whereas Study 3 resumes the longitudinal data of Study 2. Study 4 deals with time sampling data collected from a community sample using a time sampling diary (*n* = 34 couples). Finally, Study 5 is an attempt to show that the central constructs of distance and positivity are applicable to quite different dyads, namely to leaders and followers in a large Swiss organization (*n* = 91 leader–follower dyads). Sample sizes^3^ were determined by the availability of participants who were able to motivate their (dyadic) partners to concurrently answer the questionnaires. If our theoretically founded expectations are correct, the results of the five studies will support the hypothesis that distance combined with positivity are highly valid predictors of *RS.*

## Study 1

### Participants^4^ and Methods

Sixty heterosexual couples (average age 23.8 years, *SD* = 5.23), mostly students of the University of Zurich, completed the PASK5 and then indicated on 5-point scales (1 = never, 2 = rarely, 3 = sometimes, 4 = often, and 5 = almost always) (a) the frequency of negative emotions (depression, anger, disappointment, contempt, nervousness), and (b) the frequency of positive emotions (joy, sympathy, strength, liking, pride) they had experienced in their partnership during the past 2 weeks (*emotion scale* with frequencies of negative emotions reversed). In addition, they gave an overall evaluation of their relationship by answering the question “How happy is your relationship at this point in time?” from 1 = very unhappy to 6 = very happy (Hahlweg, 1996).

Based on actor–partner interdependence model (APIM) and structural equation modeling (SEM), we tested the effects of distance and positivity on *RS*. Each of the latent predictor variables has two indicators, one derived from Form *A* of the PASK5 (16 items) and the other from Form *B* (16 items with reversed coding). Latent constructs with less than three indicators often cause identification problems in measurement models which, according to Arbuckle and Wothke (1999) and Little et al. (2002), can be avoided by setting the error variances of pairs of indicators equal, thus securing their identification. We adopted this strategy.

### Results and Discussion of Study 1

**FIGURE 1:**
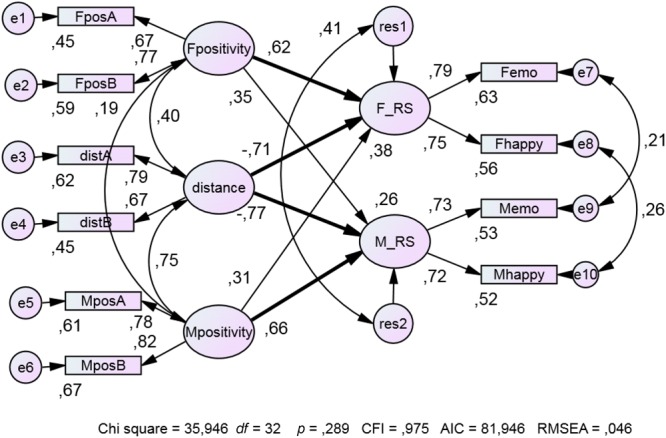
Effects of distance and positivity on relationship satisfaction in couples. Bold arrows represent significant coefficients (*p* < 0.05, one-tailed). FposA, FposB etc. are indicators of the latent predictor variables, derived from Form A and Form B of the PASK5; Fpositivity and Mpositivity are female and male positivity; distance between female and male profiles; F_RS and M_RS are female and male relationship satisfaction; Femo and Memo are female and male emotions related to the partner; Fhappy and Mhappy represent female and male happiness with the relationship (*n* = 60 couples).

**Table 1** presents descriptives and correlations of the central variables. Model *B* (**Figure 1**) differs from Model *A* (no figure) by setting the coefficients of distance as couples’ effects, the two actor effects, and the two partner effects of positivity to be equal across gender. In addition, for each of the five pairs of indicators, the error variances were set equal. Because the fit of Model *B* is not significantly poorer than that of Model *A* (Δχ^2^= 0.965; *df* = 4; *p* = 0.915 of the SPSS model comparison test), we chose the more parsimonious Model *B* as most appropriate from both a theoretical and statistical perspective.

**Table 1:** Means, standard deviations, correlations of central study variables of Study 1.

	*M*	*SD*	2	3	4	5	6	7	8	9	10
(1) FposA	5.1	2.7	0.52***	0.16	0.01	0.37**	0.25+	0.21	0.15	–0.04	0.11
(2) FposB	5.4	3.3	–	0.14	0.12	0.13	0.20	0.12	0.29*	0.06	0.24+
(3) MposA	4.2	2.7		–	0.63***	0.50***	0.42**	–0.09	–0.09	0.03	0.18
(4) MposB	5.2	2.9			–	0.49***	0.31*	–0.07	–0.03	0.10	0.19
(5) DistA	2.2	0.6				–	0.52***	–0.17	–0.04	–0.07	0.13
(6) DistB	1.9	0.5					–	–0.29*	–0.23+	–0.10	–0.18
(7) Femo	2.1	0.7						–	0.61***	0.39**	0.32*
(8) Fhappy	5.6	0.7							–	0.27*	0.41**
(9) Memo	2.0	0.7								–	0.51***
(10) Mhappy	5.4	0.7									–

*F/MPosA, female/male positivity PASK 5 form A; F/MPosB, female/male positivity PASK5 form B; DistA/B, distance between women’s and men’s personality profiles PASK5 form A/B; F/Memo, female/male frequency of positive emotions in partnership; F/Mhappy, female/male happiness in relationship; n = 60 couples; ^∗∗∗^p < 0.001, ^∗∗^p < 0.01, ^∗^p < 0.05, ^+^p < 0.10.*

As expected, the paths from distance to female (β = -0.71) and male *RS* (β = -0.77) are negative and the actor effects of female and male positivity, (β = 0.62 and β = 0.66, respectively), are positive. The partner effects of female and male positivity are positive, too, but not significant (β = 0.35 and β = 0.31). On the whole, Model *B* displays a good fit to the data (χ^2^ = 35.95, *p* = 0.289, CFI = 0.975, RMSEA = 0.046).

The pattern of regression and correlation coefficients of the independent variables reveals suppressor effects. The regression coefficients of female positivity and male positivity with female and male relationship satisfaction, respectively, are positive, whereas the regression coefficients of distance in relation to female and male relationship satisfaction, respectively, are negative. Suppressor effects are revealed by the fact that distance is positively related to positivity, but negatively to relationship satisfaction. Excluding either the distance or the two positivity variables from the model dramatically reduced the explained variance. In terms of effect sizes (of regression coefficients connecting predictors with the criteria of *RS*) the results are situated in the range of medium to large. The results of Study 1 support quite impressively the theoretical predictions: a negative relationship for distance and a positive relationship for positivity.

## Study 2

Study 2 is a replication of Study 1 if only the cross-sectional part is analyzed. We expected that distance combined with positivity would again emerge as valid negative and positive predictors of *RS*, respectively.

### Participants and Methods

#### Participants

A convenience sample was recruited among the acquaintances of students in a seminar on close relationships. There were 66 unmarried childless couples who participated at Time 1, 54 who participated also at Time 2, and 44 who participated in all three waves (with time intervals of 6 months). Women’s ages (Time 1) were between 16 and 34 (*Mdn* = 24), and men’s ages varied between 18 and 36 (*Mdn* = 26). To secure a minimum level of stability, the relationship had to have been established for at least 6 months. Recruiting only unmarried couples increased the chance of observing early stages of the relationship. The maximum duration of the partnership was 10 years, with an average duration of approximately 3 years (*Mdn* = 37 months). For women and men, respectively, the highest attained educational levels were elementary school (21 and 15%), professional school (17 and 15%), secondary school with school-leaving certificate (47 and 58%), and university degree (15 and 12%). About half of the couples (53%) were cohabiting.

#### Personality Traits

Self-ratings of the 32 items from the PASK5 on a 9-point scale provided the measures of distance and positivity (see section “Study 1”). The coefficients alpha (32 PASK5 items) for female and male positivity are 0.75 and 0.83, respectively, the coefficient alpha for distance (32 items) is 0.85. Coefficients in the range of 0.9 > *alpha* > 0.8 are generally considered to be good (see Cronbach, 1970, p. 161).

#### Relationship Satisfaction

The *RS* was assessed, as in Study 1, using (a) the *emotion scale* (see Study 1; α = 0.71), (b) the *happy time scale*: “Please think of the time you spent with your partner over the past 2 weeks. Rate the percentage of the time you felt well while you were with your partner!”, and (c) Hendrick’s (1988) *Relationship Assessment Scale* (seven items; α = 0.87 in the present sample) adapted for German by Hassebrauck (1991).

### Results and Discussion of Study 2

#### Attrition of the Sample

There were no significant differences in age or education between those couples who participated at all three points in time and those who only participated in the first or the first two waves of data collection. However, couples who dropped out after Time 1 displayed a significantly (*p* < 0.01, two-tailed) lower relationship satisfaction than those who had participated at least at Times 1 and 2. No significant differences were found between those couples who participated in the first two waves and those who participated in all three. **Table 2** presents descriptives and correlations of the central variables.

**Table 2:** Means, standard deviations, correlations of central study variables of Study 2.

	*M*	*SD*	2	3	4	5	6	7	8	9	10	11	12
(1) FposA	4.4	2.6	0.52***	0.11	0.10	0.18	–0.04	0.06	0.00	0.10	0.27*	0.22+	0.30**
(2) FposB	4.5	2.9	–	0.03	0.13	0.10	0.18	0.04	0.06	0.06	0.09	0.16	0.26*
(3) MposA	5.3	3.0		–	0.61***	0.40**	0.19	0.19	0.12	0.24+	0.08	0.20	0.32*
(4) MposB	5.4	3.6			–	0.29*	0.36**	0.31*	0.23+	0.33**	0.34	0.28	0.42**
(5) DistA	2.2	0.6				–	0.47***	–0.16	–0.20+	–0.07	–0.04	0.00	0.06
(6) DistB	2.2	0.6					–	–0.10	–0.06	–0.06	–0.09	–0.08	–0.05
(7) Femo	3.9	0.4						–	0.70***	0.74***	0.44***	0.41**	0.36**
(8) Ftime	78.8	14.9							–	0.72***	0.42***	0.47***	0.28*
(9) Fsat	5.8	8.4								–	0.54***	0.39**	0.40**
(10) Memo	4.0	3.9									–	0.57***	0.65***
(11) Mtime	81.0	14.0										–	0.73***
(12) Msat	5.7	0.8											–

*F/MPosA, female/male positivity PASK 5 form A; F/MPosB, female/male positivity PASK5 form B; DistA/B, distance between women’s and men’s personality profiles based PASK5 form A/B; F/Memo, female/male frequency of positive emotions in partnership; F/Mtime, female/male percentage of time feeling well with partner; F/Msat, female/male satisfaction with relationship; n = 66 couples; ^∗∗∗^p < 0.001, ^∗∗^p < 0.01, ^∗^p < 0.05, ^+^p < 0.10.*

Since Little’s test for data missing completely at random (MCAR) was not significant (*p* > 0.10); the SPSS 17.0 program of multiple imputation was used to estimate the missing scores on the basis of the available data under the MAR (missing at random) assumption.

#### Distance and Positivity of Self-Ratings as Predictors of *RS*

**FIGURE 2:**
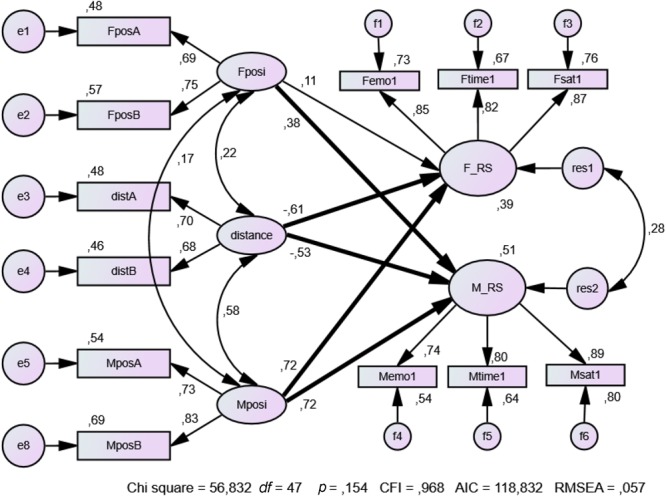
Distance and positivity influence the relationship satisfaction of couples. Bold arrows represent significant coefficients (*p* < 0.01, one-tailed). FposA is female positivity Form A. MposB is male positivity Form B. distA, distB are distances between female and male profiles derived from Form A and Form B; F_RS and M_RS are female and male relationship satisfaction; Femo1 Ftime1 Fsat1 and Memo1 Mtime1 Msat1 are female and male frequencies of positive emotions, ratio of time feeling well with the partner, and satisfaction with the relationship at Time 1 (*n* = 66 couples).

We compare Model *B* (**Figure 2**; where the parallel error variances of the predictor variables of Form *A* and Form *B* of female positivity, male positivity and distance are set equal) with Model *A* (no figure; without the constraints of Model *B*). Because the fit of Model *B* is not significantly worse than the fit of Model *A* [Δχ^2^(3) = 0.862, *p* = 0.835], we prefer Model *B* to Model *A*. The complete structural equation model fits the data quite well (Model *B*: CFI = 0.968; RMSEA = 0.057).

As predicted, the paths from distance to female (F_RS, β = -0.61) and male *RS* (M_RS, β = -0.53) are both significantly negative and the actor effects of female and male positivity, (β = 0.11 and β = 0.72, respectively), are positive but only the latter is significant. The partner effects of female and male positivity are significantly positive (β = 0.38 and β = 0.72, respectively) (**Figure 2**). Again, the combination of distance and positivity yields the most valid predictions. The positive correlations of Fpos and Mpos with distance, combined with negative coefficients of the paths from distance to F_RS and M_RS (female and male *RS*), reveal a suppressor effect of the independent variables, thus corroborating the results of Study 1. On the whole, distance combined with positivity (based on self-ratings) provides a unique and substantial contribution to the prediction of *RS*.

## Study 3

### Participants and Methods

Whereas Study 2 is an independent replication of Study 1 with data collected cross-sectionally at one measurement time, longitudinal Study 3 is an extension of Study 2. More concretely, Study 3 is based on the same sample and material as Study 2 (see Methods section of Study 2 for detailed information), but on partially different variables (i.e., partners’ ratings instead of self-ratings). Instead of rating one’s own personality, participants here rated the personality of their partner. By changing the source of the ratings, we aimed at testing whether the theoretically postulated effects generalize to different operationalizations of distance and positivity. Study 3 includes three measurement points data with missing data at Times 2 and 3 imputed (*n* = 66 couples cross-sectionally measured at Time 1 with no missing data versus *n* = 66 with *n* = 22 imputed missing data). In the longitudinal Study 3, we analyzed the intercept and slope of the *RS* data collected at three points in time (6 months interval) with missing data at Times 2 and 3 imputed.

In Study 3, we are confronted with a situation that is called *wave non-response* (Schafer and Graham, 2002, p. 150) meaning that part of the sample did not participate at all measurement occasions. Basing the analyses on the widely used procedure of listwise deletion would drastically reduce sample size. Graham (2009, p. 554) delineates various criteria for using methods imputing missing values. First, the method should yield unbiased parameter estimates over a wide range of parameters. Second, there should be a method for assessing the degree of uncertainty about parameter estimates. Third, the method should have good statistical power. The so-called full-information maximum likelihood (FIML) methods do meet these criteria and are available in SEM software like AMOS (Arbuckle and Wothke, 1999) that we used.

### Results and Discussion of Study 3 (Analysis of Change)

**Table 3** presents descriptives and correlations of the central variables. The participants of Study 3 provided data on relationship satisfaction three times. Thus, we can analyze the influence of distance and positivity on the *change* in their relationship satisfaction. Because the processes that led to initial relationship satisfaction were assumed to be similar to those that lead to further change, we expected that change would be predicted by the same variables as the initial relationship satisfaction (for different similarity effects on change in long-term marriages, see Shiota and Levenson, 2007).

**Table 3:** Means, standard deviations, correlations of central study variables of Study 3.

	*M*	*SD*	2	3	4	5	6	7	8	9
(1) cFposip	–0.9	4.8	0.12	0.15	0.07	–0.03	–0.09	0.28*	0.10	–0.01
(2) cMposip	0.9	5.9	–	0.41**	0.30*	0.28*	0.35**	0.36**	0.21+	0.32**
(3) cpappap	0.0	0.5		–	–0.13	–0.05	0.07	–0.03	–0.06	0.03
(4) Fqual1	2.9	0.4			–	0.65***	0.78***	0.52***	0.18	0.34**
(5) Fqual2	3.0	0.4				–	0.58***	0.36**	0.51***	0.30*
(6) Fqual3	2.8	0.5					–	0.30*	0.19	0.54***
(7) Mqual1	3.0	0.3						–	0.45***	0.34**
(8) Mqual2	2.9	0.4							–	0.42***
(9) Mqual3	2.8	0.5								–

*cF/Mposip, centered female/male positivity based on partner ratings; cpappap, centered distance between women’s and men’s personality profiles based on partner ratings; F/Mqual1 to F/Mqual3, female/male relationship quality at three measurement points; n = 66 couples; ^∗∗∗^p < 0.001, ^∗∗^p < 0.01, ^∗^p < 0.05, ^+^p < 0.10.*

Following Kenny et al. (2006, p. 363 f.), we tested structural equation models including the intercepts and slopes of the trajectories of *RS* as latent endogenous variables and distance together with positivity as latent exogenous variables. According to Kenny et al. (2006, p. 110), the exogenous variables female positivity (cFposip), male positivity (cMposip), and distance (cpappap) are centered at the grand mean of the variables, that is, the mean of partner ratings. In order to cover a broader variety of predictors, the distance and positivity scores are not based on self-ratings, as in Study 1 and Study 2, but on how the partner was rated. A SEM analysis based on personality self-ratings (not presented here in detail) led to a very similar structure of the coefficients.

The indicators of intercept and slope are the equally weighted averages of the three scales of relationship satisfaction (emotion scale, happy time scale, and Relationship Assessment Scale; see above), each measured three times. Their internal consistency coefficients are in the fully acceptable range of 0.73 < α < 0.91.

**FIGURE 3:**
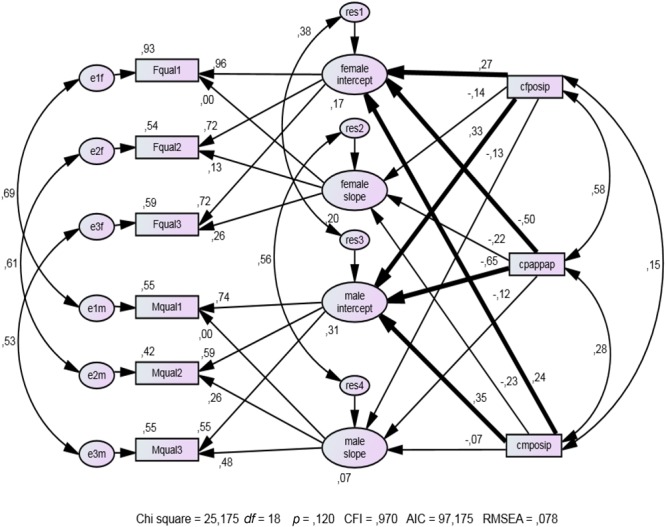
Structural equation model for predicting the intercept and slope of *RS*. Bold arrows represent significant coefficients (*p* < 0.01, two-tailed). Fqual1 to Fqual3 and Mqual1 to Mqual3 are female and male relationship quality at Times 1–3; cFposip and cMposip are female and male positivity, based on partner ratings; cpappap is the distance between female and male profiles, based on how the partner is rated (*n* = 66 couples).

We tested Model *B* (**Figure 3**) for which we set the paths from distance to both intercepts and both slopes and the paths from female and male positivity to the female and male intercepts equal across gender, against Model *A* (no figure) without all these constraints. The correlations of the corresponding error and residual scores and the correlations of observed independent variables are treated as free parameters in both models. The fit of Model *B* is not significantly poorer than that of Model *A* [Δχ^2^(6) = 9.372; *p* = 0.154], thus justifying our preference for Model *B* (Model *B*: CFI = 0.970; RMSEA = 0.078).

The distance variable (cpappap) significantly negatively predicted female and male intercepts (β = -0.50 and β = -0.65, respectively). The paths between the distance variable and female and male slopes were negative but not significant (β = -0.22 and β = -0.12, respectively). In contrast, female (cfposip) and male (cmposip) positivity on female/male intercepts (actor effects) were significantly positive (β = 0.27 and β = 0.35, respectively). Female (cfposip) and male positivity (cmposip) on female/male slopes (actor effects) were negative and not significant (β = -0.14 and β = -0.07, respectively). The partner effects of female and male positivity on intercepts are positive and significant (β = 0.33 and β = 0.24). The partner effects of female and male positivity on slopes are negative and not significant (β = -0.13 and β = -0.23). The positivity variables have positive coefficients for intercept and negative coefficients for slopes. Our assumption that initial *RS* and *RS change* would be influenced by positivity and distance in a similar way is not supported by the data.

Participants with a high degree of positivity tend to see a decrease in *RS* in the course of 12 months. This could mean they have higher, but unrealistic perceptions and expectations at the beginning which lead to a drop in *RS*. Regression toward the mean may also partly explain the opposite signs of the coefficients in predicting intercept versus predicting slope. Finally, initial *RS* has a longer history, which may have been partly influenced by different traits to the short-term changes later in the partnership (see Shiota and Levenson, 2007, for more reasons why *intercept* and *slope* can be influenced by different traits).

## Study 4

Within a longitudinal design, Study 4 uses the same combination of predictor variables as Studies 1, 2, and 3, but completely different measures of relationship satisfaction collected with the Time Sampling Diary (Brandstätter, 2007), thus excluding common methodological artifacts.

### Participants and Methods

A community sample of 34 married couples varying in age between 20 and 59 years participated in Study 4, with 35.9 and 39.8 years as the average age of wives and husbands, respectively. The education level varied between 20% elementary school, 30% apprenticeship, 20% high-school diploma, and 30% college or university degree. On 28 consecutive days, four times a day, at randomly selected times (programmed on a special wristwatch), participants recorded their own and, if present, their partner’s mood (-1, 0, and +1) and their partner’s presumable affection for the participant (9-point scale), in addition to other characteristics of the situation which were only relevant to a prior study (Wagner and Brandstätter, 1994).

Measures of *RS* were derived from the following three time sampling variables, referring to a target person’s state of mind as described by self-ratings or by partner ratings: (a) the wife’s mood as judged by the husband (component of the wife’s *RS*) and the husband’s mood as judged by the wife (component of the husband’s *RS*), (b) the wife’s affection for the husband as perceived by the husband (component of the wife’s *RS*) and the husband’s affection for the wife as perceived by the wife (component of the husband’s *RS*), and (c) the wife’s mood in the presence of the husband (component of the wife’s *RS*) and the husband’s mood in the presence of the wife (component of the husband’s *RS*).

Aggregating the records separately over the first, second, and third part of equally frequent consecutive observations led to a time series of measures of *RS* with coefficients α = 0.54, α = 0.65, and α = 0.44 for Time 1, Time 2, and Time 3. Again, we applied the model of **Figure 3** with intercepts and slopes of *RS* as dependent variables. Distance and positivity as independent variables are based on self-ratings. No partner ratings were collected in this study.

### Results and Discussion of Study 4

**FIGURE 4:**
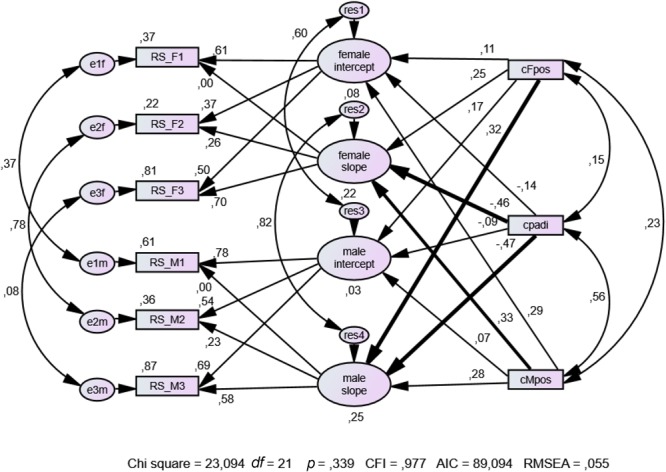
Influence of distance and positivity on relationship satisfaction based on time-sampling data. Bold arrows represent significant coefficients (*p* < 0.05, one-tailed). cFpos is female and male positivity; cpadi is distance between female and male profiles; *RS_F1, RS_F2 RS_F3, RS_M1, RS_M2*, and *RS_M3* are female and male relationship satisfaction at Time 1, Time 2, and Time 3 (*n* = 34 couples).

**Table 4** presents descriptives and correlations of the central variables. We compared Model *B* (**Figure 4**; residual variances and β-coefficients predicting *RS* set equal across gender) with Model *A* (no figure; without any restraints) and opted for Model *B* as most adequate in terms of theoretical considerations and empirical fit, which is not significantly different from the fit of Model *A* (Δχ^2^= 8.457; *df* = 8; *p* = 0.390). On the whole, Model *B* displays a good fit to the data (CFI = 0.977, RMSEA = 0.055).

**Table 4:** Means, standard deviations, correlations of central study variables of Study 4.

	*M*	*SD*	2	3	4	5
(1) Fpos	15.1	7.1	0.23	0.15	0.05	–0.05
(2) Mpos	14.7	7.6	–	0.56**	0.22	0.07
(3) padi	2.6	0.7		–	–0.09	–0.19
(4) F_RS	3.7	0.3			–	0.66***
(5) M_RS	3.7	0.3				–

*F/Mpos, female/male positivity; padi, distance between women’s and men’s personality profiles; F/M_RS, female/male relationship satisfaction averaged across three measurement points; n = 34 couples; ^∗∗∗^p < 0.001, ^∗∗^p < 0.01, ^∗^p < 0.05, ^+^p < 0.10.*

As expected, both paths from distance (cpadi) to female slope and male slope are negative and significant (β = -0.46 and β = -0.47). The β-coefficients of distance predicting the female and male intercepts are in the expected direction, but not statistically significant (β = -0.14 and β = -0.09). The same applies to *actor effects* of female (cFpos) and male (cMpos) positivity that are in the expected direction but not significant predicting the female and male slopes (β = 0.25 and β = 0.28) and female and male intercepts (β = 0.11 and β = 0.07), respectively. With respect to *partner effects*, female positivity is significantly related to male slope (β = 0.32) but (not significantly) related to male intercept (β = 0.17). Further, male positivity is significantly related to female slope (β = 0.33) but (not significantly) related to female intercept (β = 0.29). The distance effects of Study 4, as those of Study 3, are – with some variation in the size of the coefficients – negative on all four dependent variables. Contrary to Study 3, where the effects of positivity are positive on intercept and negative (close to zero) on slope, in the present study all positivity effects are (some significantly) positive. Moreover, intercept and slope are predicted by the same variables (distance throughout with negative signs and positivity throughout with positive signs), but not with the same weights.

## Study 5

In Study 5, we ask whether the effects of distance and positivity of personality profiles found in Studies 1–4 can be replicated with supervisor-subordinate dyads. In general, their relationship is less close and less emotionally accentuated, but mutual understanding and trust as well as ease of cooperation are also thought to depend on the similarity of their personality profiles. One of the most important positive outcomes of leader–follower interactions are satisfaction with the mutual (follower–leader) relationship, on the one hand, and job satisfaction, on the other hand (e.g., Hackman and Oldham, 1975; Graen and Uhl-Bien, 1995; Krumm et al., 2013). Study 5 is meant to contribute to the generalizability of the results beyond the field of intimate partnerships.

Zhang et al. (2012) were able to show that leader-follower congruence in measures of proactive personality (characterized by taking the initiative in improving one’s life circumstances and applying new methods of problem solving) is a condition of well-functioning leader-member exchange (LMX), which in turn promotes workers’ job satisfaction, affective commitment, and job performance. We expected comparable effects for similarity in the PASK5 profiles, in particular when combined with the positivity of the profile.

Leader–follower similarity is assumed to facilitate agreeing on goals, perceiving the chances of or obstacles toward goal achievement, and coordinating efforts. Moreover, similarity contributes to mutual liking, thus securing mutual respect and rewarding social interaction. Combining distance and positivity as independent variables in Study 5 should again reveal a suppressor effect that increases the percentage of explained variance well beyond the sum of the percentages explained by each predictor on its own.

### Participants and Methods

The participants of Study 5 were 91 leaders (29 women) and 91 followers (53 women). On average, the leaders were 45 (*SD* = 8.6), the followers 42 (*SD* = 12.5) years old. The participants were recruited by (a) sending a link from our study website to the human resources representative of a leading Swiss insurance company, and (b) the human resource manager sending e-mails (with a link to our website) to 420 German-speaking managers who regularly interacted with their subordinates. Having answered their own questionnaires, the supervisors invited a strictly randomly selected follower by e-mail (with a shared code for dyad identification and a link to our study website) to participate in the study. As an acknowledgment, the leaders and followers received a report of the general results and a book after the end of the study. Among other questionnaires, which are not relevant in the present context, the participants answered Form *A* of the PASK5 as a self-rating questionnaire, an adaptation of Hassebrauck’s (1991) relationship satisfaction scale for the leader–follower context with seven items, α = 0.80 (leader) and α = 0.85 (follower), and the Job Diagnostic Survey of Hackman and Oldham (1975) in German as a measure of job satisfaction, with an acceptable internal consistency of the 5-items, α = 0.63 (leader) and α = 0.73 (follower), in the present sample.

### Results and Discussion of Study 5

**FIGURE 5:**
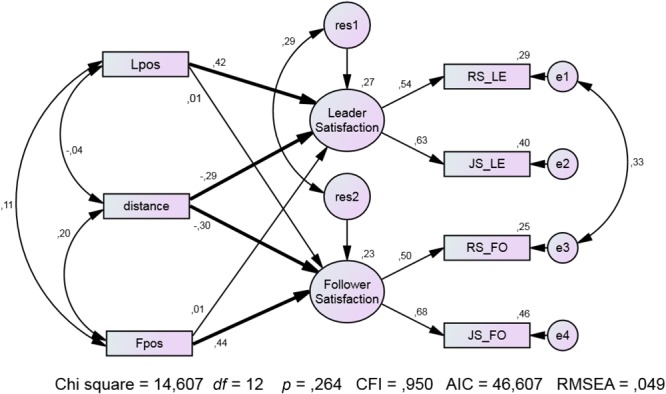
Relationship and job satisfaction depending on similarity and positivity of personality profiles in leader–follower dyads. Bold arrows represent significant coefficients (*p* < 0.05, one-tailed). Lpos and Fpos are leader and follower positivity; distance is the distance between leader and follower personality profiles; RS_LE and RS_FO are leader and follower relationship satisfaction; JS_LE and JS_FO are leader and follower job satisfaction. Bold arrows represent significant coefficients (*p* < 0.05, one-tailed) (*n* = 91 dyads).

**Table 5** presents descriptives and correlations of the central variables. With Model *B* (**Figure 5**), the two actor effects, the two partner effects, the two distance effects, and the residual variances were set equal across the social roles (leader and follower). In addition, the error variances of both indicators of leader satisfaction and follower satisfaction, respectively, were set equal. In contrast, Model *A* (no figure) does not have these constraints. Because the fit of Model *B* was not significantly poorer than the fit of Model *A* (Δχ^2^ = 4.638; *df* = 6; *p* = 0.591), we preferred Model *B* which reveals significant actor effects for leaders’ and followers’ positivity as well as significant negative effects for distance.

**Table 5:** Means, standard deviations, correlations of central study variables of Study 5.

	*M*	*SD*	2	3	4	5	6	7
(1) LPos	6.3	3.4	0.11	–0.04	0.25*	–0.08	0.23*	0.08
(2) FPos	5.9	3.5	–	0.20+	0.07	0.26*	0.02	0.23*
(3) Distance	1.8	0.5		–	–0.10	–0.03	–0.19+	–0.22*
(4) RS_LE	6.1	0.6			–	0.29**	0.19+	0.25*
(5) RS_FO	6.1	0.8				–	–0.02	0.48***
(6) JS_LE	5.6	0.8					–	0.15
(7) JS_FO	5.3	0.8						–

*L/FPos, leader/follower positivity; Distance, distance between leaders’ and followers’ personality profiles; RS_LE/FO, leader/follower satisfaction with relationship to follower/leader; JS_LE/FO, leader/follower job satisfaction; n = 91 leader–follower dyads; ^∗∗∗^p < 0.001, ^∗∗^p < 0.01, ^∗^p < 0.05, ^+^p < 0.10.*

Only Form *A* of the PASK5 was answered in this study, preventing the use of two parallel indicators of the latent constructs. Therefore, distance and positivity are represented in **Figure 5** as observed variables.

A person’s positivity score is positively correlated with her/his own relationship satisfaction (β_leader_ = 0.42 and β_follower_ = 0.44), but not with the partner’s relationship satisfaction (both βs = 01; **Figure 5**). When the actor and partner effects are in addition set equal, the fit deteriorates drastically (Δχ^2^ = 23-405; *df* = 4; *p* = 0.000) proving that the actor-partner difference is highly significant. In Study 5, the actor effects are much stronger than the partner effects. This could mean that the leader–follower dyads are socially less closely interconnected than the couples in Studies 1–4.

As expected, the β-coefficients of distance predicting the leader’s and the follower’s satisfaction are significantly negative (β_leader_ = -0.29 and β_follower_ = -0.30). The pattern of correlations and regression coefficients can be interpreted as a small (not significant) suppressor effect which is illustrated by comparing the regression coefficients of distance (in predicting, for instance, follower satisfaction) before (β = -0.12) and after (β = -0.18) including positivity scales (leader’s positivity, Lpos, and follower’s positivity, Fpos) in the regression model. More important, as to be seen in **Figure 5**, the paths to leader satisfaction and follower satisfaction are significant; because of the negative weight of distance and the positive weight of Lpos and Fpos, respectively, there is a suppressor effect statistically asserted.

## General Discussion

Our five studies share distance (i.e., dissimilarity) between and positivity of item-based personality profiles of two individuals in a dyad (romantic partners or leader–follower dyads) as predictor variables and *RS* as the criterion variable. Whereas the predictor variables (distance and positivity) are the same across studies, the measures of *RS* are partially different.

### Distance as a Predictor of *RS*

In each of the five studies, distance is a significant negative predictor of the partners’ *RS*, particularly when the models include positivity of their self-rated personality profiles, defined by the 32 items of the PASK5. In contrast to the contradictory results reported in the literature, the importance of global distance (average across 32 items of absolute differences between the two partners’ personality self-ratings) for *RS* has been unequivocally corroborated.

### Positivity as a Predictor of *RS*

The global variable positivity is a valid predictor of *RS*, too, in particular when it is combined with distance. Positivity absorbs the valence aspects of the 32 items of the PASK5, indirectly also those of the Big Five. Furr (2008) reports high correlations between the average individual profile and the social desirability profile of expert ratings. Our positivity scale is highly correlated with the profile similarity between the individual profile and the average profile. Focusing on the effects of global distance and global positivity, we have not delved into narrower domains of similarity as, for instance, Zentner (2005) did for the domains of the Big Five.

Positivity (*P*) means positive self-rating, that is, a form of generalized attitude of individuals concerning their interaction with the social surrounding. It is often but not in general conceptually connected with positive relationship as central for relationship satisfaction (*RS*). Consequently, it is positive correlated with *RS* but distinctively different from it. It is also connected with distance of self-rating (*DS*) of two partners, meaning that a higher positivity reduces the distance to all (important) partners.

We already could show that the effects of positivity and distance are not restricted to the specific form of measuring these constructs. Substituting self-ratings by partner-ratings in Study 3 leads to very similar results: positive effects on *RS* of positivity and negative effects of distance. A further argument for the generalizability of the positivity construct is given by the fact that defining positivity in terms of the two most extreme scores (1, 9) only did not affect the results. In any case, the reciprocal suppressor effects of the two predictor variables were found again.

### Suppressor Effects

It will be remembered that a reciprocal suppressor effect is revealed when adding a second predictor (e.g., positivity) increases the coefficient of the first (e.g., distance) and vice versa. This implies that the zero order correlation between predictor *X* and criterion *Z* is lower than the partial correlation between *X* and *Z*, when controlling for the second predictor *Y* (Maassen and Bakker, 2001). Having found suppressor effects for distance and positivity in the pilot study, we had expected that combining distance with positivity in each of the five studies would also lead to suppressor effects, which should result in a higher predictability of *RS* than that reported in recent publications (Barelds, 2005; Luo et al., 2008; Dyrenforth et al., 2010; Furler et al., 2013). Our expectations are clearly supported by the data. The seemingly high validity of the distance-positivity model of *RS* might call for special care in interpreting the construct validity of the latent variables, possibly under the premise: “It is too good to be true.” However, discounting the scientific value of a model because the effect sizes are astoundingly (unbelievably) high would seem to be the wrong conclusion.

It is worth noting that controlling for the main effects of personality traits is usually associated with smaller effects of similarity (Dyrenforth et al., 2010). However, the opposite is true for distance and positivity in our studies. Obviously, contrary to past empirical evidence, the constructs distance and positivity have some unique features which increase their predictive validity and which may be relevant beyond the field of relationship satisfaction.

For a better understanding of the suppressor effects, we must realize – something we did not appreciate at the beginning of our analyses – that a positive correlation between distance and positivity follows from the very construction of the distance and positivity scales: the more extreme (i.e., positive) a partner’s ratings are, the more room there is for high distance scores if the positivity scores of the two partners are only moderately correlated, as is the case in our studies. Note that with different operationalizations of distance (e.g., communicator’s posture, Mehrabian, 1968; similarity in person perception, Alves et al., 2016; psychological distance of an event in terms of time or space, Van Boven et al., 2010) there is a negative correlation between distance and variables of positivity (such as positive attitudes, liking). In our studies, the positive correlation between distance and positivity (due to the specific operationalization) implies a suppressor effect, when *RS* is negatively correlated with distance and positively with positivity.

### Actor and Partner Effects

In the first and, most pronounced, in the fifth study, the actor effects are stronger than the partner effects (see Watson et al., 2004; Donnellan et al., 2007; Luo et al., 2008; Dyrenforth et al., 2010; Furler et al., 2013 for similar results). One may assume that the members of the leader–follower dyads are less mutually dependent than the members of the couples in the other studies. Actor effects may also be stronger since cause and effect remain within the person, while partner effects are socially transactional (cf. Zentner, 2005; Kenny et al., 2006, p. 149/150). Nevertheless, actor effects are not generally stronger than partner effects, as is shown by Studies 3 and 4. Thus, future research needs to clarify the conditions that make actor or partner effects relatively more important.

### Predicting the Intercept and Slope of *RS* in Longitudinal Designs

We found that both aspects of *RS* (intercept and slope) can be predicted by distance and positivity, albeit partly with different weights and, in the case of positivity, even with different signs. In our studies, the intercept is the estimated *RS* status at the beginning of observation (time zero). One must take into consideration that the relationships already have a more or less extended history at time zero. It seems plausible that the intercept and slope should call for somewhat different explanations. Future studies may collect retrospective information on the history of the relationship in order to better understand its development from the first date till time zero of the study.

### Using the Format of the Scales for Other Kinds of Studies

Going beyond partnership research, the concepts of distance and positivity can be applied whenever the profile of an individual is matched with the profile of a group or of an environment’s demands, as we have seen in the pilot study. The person-environment fit, represented by a small distance between two personality profiles, can be an important condition for well-being and achievement. The *distance-positivity model* has the potential to serve as a remarkable improvement in predicting similarity effects of various kinds.

In designing questionnaires with personality descriptive adjectives or behavioral statements, one generally tries to avoid items with explicit valence connotations. We guess, however, that the combination of distance and positivity works best when the items have a moderate valence connotation connected with bipolar items like those of the PASK5.

The *distance-positivity model of RS* maintains its validity when self-ratings are substituted by partner ratings (Study 3). Although comparing the predictive validity of various measures of distance and positivity and of various perspectives (self- or partner perspective) was not our primary goal, there is some empirical evidence suggesting that the benefits of suppressor effects in predicting *RS* are linked to the type of ratings, in that partner ratings somewhat outperformed the predictive validity of self-ratings (with *RS* as criterion).

Although many questions still remain open, we expect that *distance* combined with *positivity* will prove useful beyond the field of dyadic relationship research (Karney and Bradbury, 1995). Analyzing profiles that are based on the 32 items of the PASK5 and not, as is more common, on the scales of the Five Factor Model, forgoes exploring the connection between personality types and *RS* (see, for example, Altmann et al., 2013). However, being aware of the limits imposed by the small sample sizes, which may render complex models particularly problematic, we chose distance and positivity of personality profiles as the minimum number of theoretically meaningful explaining variables. Choosing and specifying models not exclusively *a priori*, but to some extent *post hoc* on the basis of fit indices, as we did, bears some risk of capitalizing on chance. However, the consistent replication of the basic results in five studies is a strong argument for trusting our findings.

## Conclusion

There is no theoretically simple way of construing or detecting reciprocal suppressor variables. In our case, it was a serendipitous finding of the pilot study that was replicated in five studies using four independent samples. Nevertheless, the suppressor effects of distance and positivity are in need of further statistical and psychological clarification. Our studies are a first step in that direction, primarily toward establishing the predictive validity of distance and positivity. Looking more closely at the inter- and intra-personal processes by which personality traits influence *RS* in dyads (see, for instance, Donnellan et al., 2004; Hampson, 2012) will be of primary importance.

A promising strategy for future relationship research might be to first construct formal models simulating the process that generates events of interpersonal reward and punishment, with probabilities that are a function of distance and positivity of their personality profiles. Step by step, the models could become more complex (and more realistic) by including fictitious parameters of possibly facilitating or obstructing life circumstances and events, before a process model is tested empirically.

## Author Contributions

HB developed theoretical idea, collected data, performed statistical analyses, and wrote up the article. VB developed theoretical idea, collected data, and wrote up parts of the article. RP gave statistical advice and commented on the article.

## Conflict of Interest Statement

The authors declare that the research was conducted in the absence of any commercial or financial relationships that could be construed as a potential conflict of interest.

## Figures and Tables

**FIGURE 0:**
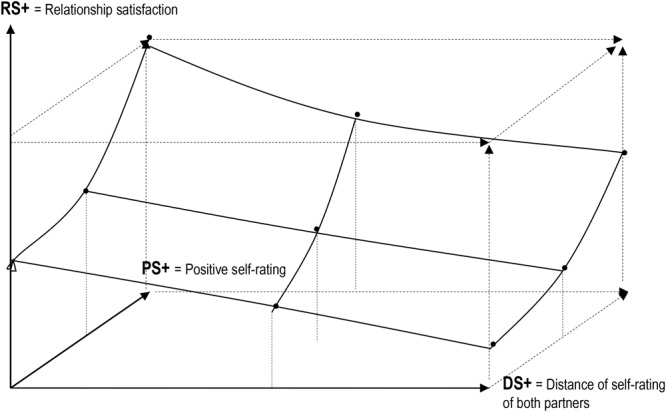
Theoretical model of relationship satisfaction (*RS*) as function of distance of self-rating of both partners (*DS*) (monotonically decreasing) and Positive self-rating (*PS*) (monotonically increasing). Based on this model, *RS* is high (low), if *PS* is high (low), and *RS* is low (high), if *DS* is high (low). Disturbing of clear relation is a negative correlation between *PS* and *DS* and possibly further suppressor effects of other variables as socio-economic status, health, interests, partnership model (culture) etc.

## Footnotes

1 Comparing various measures of positivity, though relevant for clarifying its construct validity, was beyond the scope of the present studies.

2 Indeed, theoretically it is well conceivable to define positivity in terms of the two most extreme scores (1, 9) only. From a methodological point of view, though, considering the four most extreme scores (1, 2, 8, 9) seems advisable as the variance in positivity is less restricted which renders the positivity measure more reliable and valid. The higher validity of the more comprehensive positivity measure is exemplarily illustrated in Study 2 in terms of the correlations between positivity and *RS*: *r*(132) = 0.26 (comprehensive measure) versus *r*(132) = 0.16 (less comprehensive measure). In any case, the results are highly comparable.

3 Sample sizes were determined by the availability of participants who were able to motivate their (dyadic) partners to concurrently answer the questionnaires. As thumb rule for statistical stability for multivariate methods like SEM (structural equation modeling) is accepted the following relation: N:m should be >3, *N* = sample size, *m* = number of variables. This condition is all but realized in all five studies.

4 All the reported studies were carried out in accordance with the recommendations of APA ethical guidelines for empirical psychological research with human participants. All participants gave written informed consent and were debriefed about the purpose of the study after the data collection period. Most important, Studies 1 and 5 were conducted at the University of Zurich where researchers, before conducting a study, have to fill out a checklist on a variety of ethically relevant aspects of the study. If all the mentioned ethical hazards (e.g., deception, questions on sexuality, danger to one’s psychological or physical integrity) can be turned down, no formal approval of the local ethics committee is required – which was the case for Studies 1 and 5. In contrast, Studies 2, 3, and 4, were conducted at the University of Linz where, at the time of data sampling, no formal ethics committee for psychological research existed. Researchers were forced to comply with APA ethical standards when conducting research and publishing it.

## APPENDIX

Theoretical concept of the (stochastic) function between *RS* (= relationship satisfaction) as criterion and *DS* (= distance of self-rating [of both partners]) at one hand and *PS* (= measure of positivity of self-rating) on the other hand (cf. Figure below) is a non-linear but monotone function in the trivariate model *RS* = *f*(*DS,PS*): (a) The effect of *DS* = distance of self-rating on *RS* is negative because of growing misunderstanding, growing annoyance due to different interests, but reducing with growing distance, because it is losing its importance. In the same way, little *DS* avoids many situations of possible complications between the partners. (b) The effect of *PS* = positivity of self-rating on *RS* is positive because of expecting a good relation to most people, especially to the partner. This premature praise will open the heart of the partner for a good relationship and doing the same with him. On the other side, little *PS* will reduce the possibilities of positive evaluation of the actions of others, especially the partners. So it is plausible to expect a function among the three variables as shown in the figure 0 below.
